# The Central Role of Liver Function at Treatment Initiation and Its Preservation at Progression for Post-Progression Survival After Atezolizumab Plus Bevacizumab in Advanced Hepatocellular Carcinoma

**DOI:** 10.3390/biomedicines14010232

**Published:** 2026-01-21

**Authors:** Mizuki Ariga, Teiji Kuzuya, Hisanori Muto, Yoshihiko Tachi, Mariko Kobayashi, Hijiri Sugiyama, Sayaka Morisaki, Gakushi Komura, Takuji Nakano, Hiroyuki Tanaka, Kazunori Nakaoka, Eizaburo Ohno, Kohei Funasaka, Mitsuo Nagasaka, Ryoji Miyahara, Yoshiki Hirooka

**Affiliations:** 1 Department of Gastroenterology and Hepatology, Fujita Health University, 1-98 Dengakugakubo, Kutsukake-Cho, Toyoake 470-1192, Aichi, Japan; mizuki.ariga@fujita-hu.ac.jp (M.A.); hisanori.muto@fujita-hu.ac.jp (H.M.); ytachi@fujita-hu.ac.jp (Y.T.); mariko.kobayashi@fujita-hu.ac.jp (M.K.); hijiri.sugiyama@fujita-hu.ac.jp (H.S.); sayaka.kameshima@fujita-hu.ac.jp (S.M.); gakushi.komura@fujita-hu.ac.jp (G.K.); tkjnkn@fujita-hu.ac.jp (T.N.); hiroyuki.tanaka@fujita-hu.ac.jp (H.T.); knakaoka@fujita-hu.ac.jp (K.N.); eizaburo.ono@fujita-hu.ac.jp (E.O.); k-funa@fujita-hu.ac.jp (K.F.); nmitsu@fujita-hu.ac.jp (M.N.); ryoji.miyahara@fujita-hu.ac.jp (R.M.); yoshiki.hirooka@fujita-hu.ac.jp (Y.H.); 2 Department of Gastroenterology and Hepatology, Fujita Health University Bantane Hospital, Nagoya 454-8509, Aichi, Japan; 3 Department of Gastroenterology, Fujita Health University Okazaki Medical Center, Okazaki 444-0827, Aichi, Japan

**Keywords:** hepatocellular carcinoma, immunotherapy, atezolizumab, bevacizumab, liver function, post-progression survival

## Abstract

**Background/Objectives**: Atezolizumab plus bevacizumab (Atz+Bev) is widely used for advanced hepatocellular carcinoma (HCC), yet predictors of post-progression survival (PPS), a clinically meaningful endpoint reflecting the feasibility of treatment sequencing, remain unclear. We aimed to identify determinants of PPS and factors associated with successful transition to subsequent therapy after progressive disease (PD) on Atz+Bev. **Methods**: We retrospectively analyzed 132 patients with HCC who initiated Atz+Bev with Child–Pugh A and Eastern Cooperative Oncology Group performance status (ECOG PS) 0/1. PPS was defined as survival from radiological PD to death; tumor response was assessed by RECIST v1.1. **Results**: Among 132 patients treated with Atz+Bev, median progression-free and overall survival were 9.2 and 21.2 months. PD occurred in 97 patients, with a median PPS of 9.2 months. At PD, 76 patients (78.4%) maintained both Child–Pugh A and ECOG PS 0/1; 93.4% of these patients transitioned to subsequent therapy, compared with 38.0% of patients who did not maintain Child–Pugh A and ECOG PS 0/1. The median PPS values were 14.7 and 2.0 months, respectively (*p* < 0.0001). In this PD cohort, disease control achieved with subsequent therapy after radiological PD was associated with longer PPS (16.1 vs. 5.0 mosnths; *p* = 0.0002). ECOG PS 0, Child–Pugh A, absence of portal vein invasion, and AFP < 400 ng/mL at PD independently predicted prolonged PPS. A baseline Child–Pugh score of 5 independently predicted preservation of Child–Pugh A and ECOG PS 0/1 at PD. **Conclusions**: Initiating Atz+Bev under optimal liver function (Child–Pugh 5) and preserving hepatic reserve and performance status through progression are critical for enabling subsequent therapy and achieving longer PPS.

## 1. Introduction

The treatment strategy for advanced hepatocellular carcinoma (HCC) has undergone a substantial transformation with the advent of immune checkpoint inhibitor (ICI)-based combination therapies [1,2,3]. Several ICI-containing regimens, including atezolizumab plus bevacizumab (Atz+Bev) [4], durvalumab plus tremelimumab [5], and nivolumab plus ipilimumab [6], are now established as first-line treatment options. As a result, the management of advanced HCC has shifted toward a strategy that assumes sequential administration of multiple effective systemic therapies [7,8]. In addition, multiple molecular targeted agents are now available, further emphasizing the importance of treatment sequencing in which therapies are continuously transitioned even after failure of initial treatment.

With this paradigm shift, overall survival (OS) is no longer determined solely by the antitumor efficacy of first-line therapy. Rather, the ability to maintain clinical eligibility and transition to subsequent treatment after radiological disease progression has emerged as a critical determinant of long-term outcomes [7,8]. In this context, post-progression survival (PPS), defined as survival after progressive disease (PD), has gained attention as a clinically meaningful endpoint that reflects the feasibility of treatment sequencing rather than the efficacy of a single regimen [7]. However, despite its increasing relevance in the immunotherapy era, PPS has rarely been evaluated as a primary outcome. Although several studies have reported outcomes of individual subsequent therapies, the clinical factors that enable patients to maintain treatment eligibility through progression and thereby achieve prolonged PPS remain insufficiently defined.

Preservation of liver function is a prerequisite for systemic therapy in HCC, and Child–Pugh class A is widely used as a criterion for treatment eligibility in both clinical trials and routine practice [1,2,3]. Nevertheless, Child–Pugh class A represents a heterogeneous population. In particular, the clinical significance of initiating treatment with a Child–Pugh score of 5, as distinct from a score of 6, has not been systematically examined with respect to preservation of hepatic reserve and performance status (PS) at the time of PD nor has its impact on subsequent treatment eligibility and PPS.

We hypothesized that initiation of Atz+Bev under optimal liver function—specifically, a Child–Pugh score of 5—plays a pivotal role in preserving hepatic reserve and PS until PD. Such preservation would facilitate transition to subsequent therapy and ultimately result in prolonged PPS. To test this hypothesis, we conducted a retrospective real-world analysis focusing on PPS as the primary outcome in patients with advanced HCC treated with Atz+Bev. We evaluated liver function and clinical status at both treatment initiation and radiological progression to clarify the determinants of treatment eligibility after PD and their association with PPS.

## 2. Materials and Methods

### 2.1. Patients

Between October 2020 and March 2025, a total of 150 patients with HCC classified as Barcelona Clinic Liver Cancer (BCLC) stage C or as BCLC stage B disease considered unsuitable for further locoregional therapies such as hepatic resection, radiofrequency ablation, or trans-arterial chemoembolization (TACE) were initiated on Atz+Bev at our institution. Eighteen patients were excluded due to Child–Pugh B liver function or an Eastern Cooperative Oncology Group performance status (ECOG PS) of 2 at treatment initiation. The remaining 132 patients who met both criteria—Child–Pugh A and ECOG PS 0 or 1 (CP-A/PS-0/1)—were retrospectively analyzed to evaluate treatment outcomes.

All study procedures were approved by the Ethics Committee of Fujita Health University School of Medicine (approval No. HM24-418) and conducted in accordance with the principles of the 1975 Declaration of Helsinki. Written informed consent for Atz+Bev therapy was obtained from all patients. The need to obtain informed consent for inclusion in this study was waived by the Ethics Committee based on the retrospective nature of the analysis.

### 2.2. Atz+Bev Treatment and Evaluation of Adverse Events (AEs)

All patients received intravenous atezolizumab 1200 mg and bevacizumab 15 mg/kg every three weeks [4]. AEs were evaluated according to the Common Terminology Criteria for Adverse Events version 5.0. In cases of treatment-related AEs of grade 3 or higher, either or both drugs were temporarily withheld until the event improved to grade ≤ 2, in accordance with manufacturer-recommended guidelines. Atz+Bev treatment was continued until a potentially life-threatening AE occurred or clinical or radiological disease progression was confirmed.

### 2.3. Evaluation of Radiological Antitumor Response

Radiological antitumor response was assessed using Response Evaluation Criteria in Solid Tumors version 1.1 (RECIST v1.1) by experienced hepatologists. Dynamic contrast-enhanced computed tomography was performed at baseline, six weeks after treatment initiation, and every 4–12 weeks thereafter. Disease control (DC) was defined as complete response (CR), partial response (PR), or stable disease (SD), whereas non-disease control (non-DC) was defined as PD or not evaluable (NE). Radiological PD during Atz+Bev was defined as the first RECIST v1.1-confirmed progression. After PD, antitumor response to subsequent therapy was evaluated independently using RECIST v1.1, with imaging at the time of PD serving as the reference baseline.

### 2.4. Subsequent Treatment

Subsequent treatment was defined as any anticancer therapy administered after radiologically confirmed PD, including systemic therapy and/or locoregional therapy and, when applicable, continuation of Atz+Bev beyond PD, but excluding best supportive care (BSC). Patients managed with BSC alone after radiologically confirmed PD were classified as not having received subsequent treatment.

### 2.5. Statistical Analysis

All statistical analyses were performed using EZR version 1.29 (Saitama Medical Center, Jichi Medical University, Shimotsuke-shi, Tochigi-ken, Japan) [9]. Continuous variables are presented as median and range, and categorical variables as number and percentage. Progression-free survival (PFS), OS, and PPS were estimated using the Kaplan–Meier method and compared using the log-rank test. Categorical variables were compared using Fisher’s exact test. PFS was defined as the time from initiation of Atz+Bev to progression or death, OS as the time to death or last follow-up, and PPS as the time from progression to death or last follow-up. Prognostic factors for PPS were evaluated using uni- and multivariate Cox proportional hazards models; variables with *p* < 0.05 in univariate analysis were included in the multivariate model. AFP was dichotomized at 400 ng/mL, a cut-off widely used in established prognostic models and prior HCC trials, including the CLIP score [10]. Missing data were minimal; therefore, complete-case analysis was performed. All tests were two-sided, and *p* < 0.05 was considered statistically significant.

## 3. Results

### 3.1. Baseline Characteristics (n = 132)

Baseline characteristics of the 132 patients who initiated Atz+Bev are summarized in Table 1. Median age was 75 years (range, 38–90 years), and 109 patients (82.6%) were men. Non-viral etiology was observed in 89 patients (67.4%). Atz+Bev was given as first-line systemic therapy in 94 patients (71.2%) and as second-line or later in 38 patients (28.8%). A total of 96 patients (72.7%) had a Child–Pugh score of 5, and 105 (79.5%) showed ECOG PS 0 at baseline. Median serum AFP level was 39.7 ng/mL, with 35 patients (26.5%) showing AFP ≥ 400 ng/mL The median observation period was 16.1 months (range, 0.8–53.6 months).

### 3.2. Overall Treatment Outcomes (n = 132)

**Figure 1:**
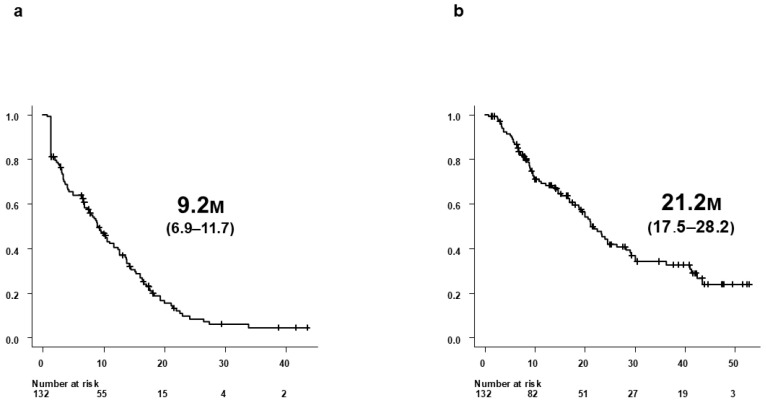
Survival outcomes of all 132 patients treated with Atz+Bev. (**a**) Progression-free survival (PFS) in all 132 patients who initiated Atz+Bev therapy. (**b**) Overall survival (OS) in the same cohort of 132 patients. Median PFS was 9.2 months (95% CI, 6.9–11.7), and median OS was 21.2 months (95% CI, 17.5–28.2).

Median PFS was 9.2 months (95% confidence interval [CI], 6.9–11.7), and median OS was 21.2 months (95% CI, 17.5–28.2) (Figure 1a,b). Median duration of Atz+Bev treatment was 7.7 months. According to RECIST v1.1, best overall antitumor response was CR in 1 patient (0.8%), and PR in 46 (34.8%), SD in 59 (44.7%), PD in 24 (18.2%), and NE in 2 (1.5%) patients. The objective response rate (ORR) and disease control rate (DCR) were 35.6% and 80.3%, respectively.

### 3.3. Clinical Outcomes and Baseline Characteristics of Patients Who Developed PD (n = 97)

**Figure 2:**
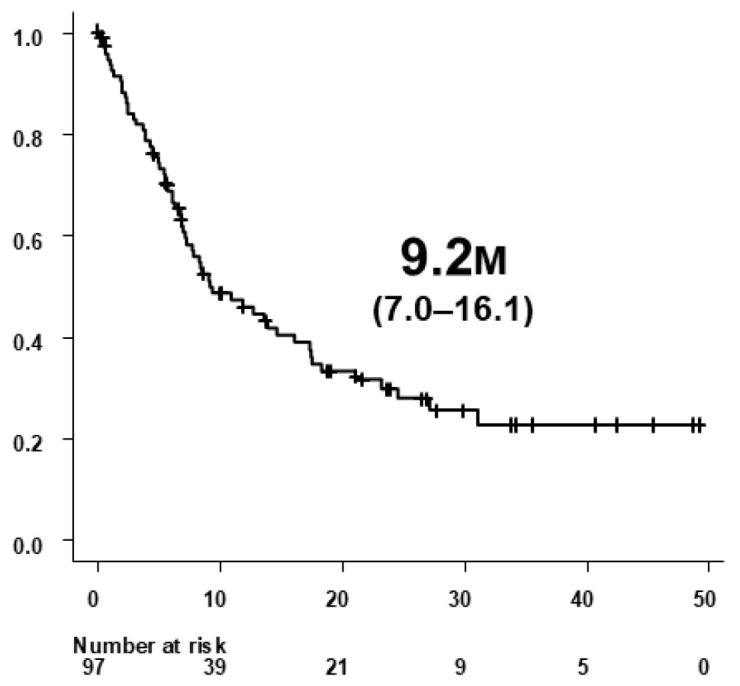
Post-progression survival (PPS) in 97 patients who developed radiologically confirmed progressive disease (PD) during atezolizumab plus bevacizumab (Atz+Bev) therapy. PPS was defined as the time from radiological confirmation of PD to death or last follow-up. The median PPS was 9.2 months (95% CI, 7.0–16.1).

Of the 132 patients treated with Atz+Bev, 97 developed radiologically confirmed PD according to RECIST v1.1. Baseline characteristics of these patients at treatment initiation are summarized in Table 1. Median PPS among the 97 patients was 9.2 months (95% CI, 7.0–16.1) (Figure 2).

### 3.4. Clinical Status at the Time of Radiological PD During Atz+Bev Therapy

**Figure 3:**
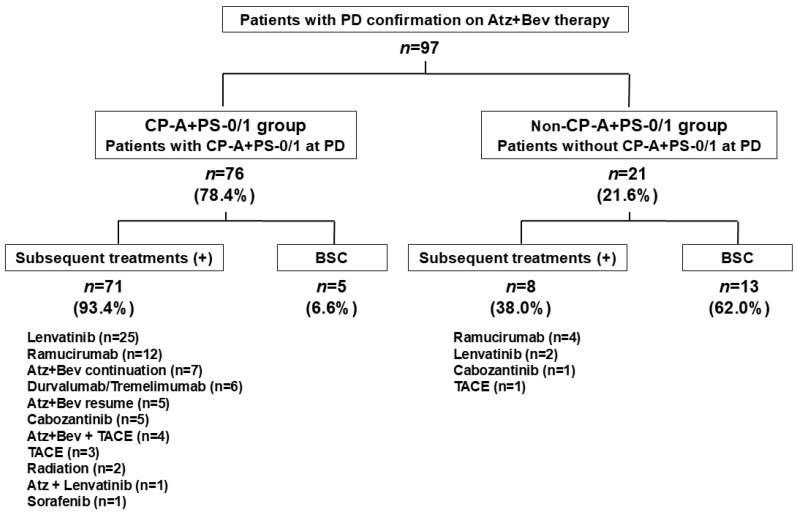
Clinical status at radiological progression during Atz+Bev therapy and subsequent treatment patterns. Among the 97 patients with radiological PD, 76 (78.4%) maintained both Child–Pugh A and ECOG performance status 0 or 1 at progression (CP-A/PS-0/1 group), while 21 (21.6%) did not (non-CP-A/PS-0/1 group). Subsequent systemic therapy was administered to 71 of 76 patients (93.4%) in the CP-A/PS-0/1 group, versus 8 of 21 patients (38.0%) in the non-CP-A/PS-0/1 group. Best supportive care (BSC) was selected in 6.6% of the CP-A/PS-0/1 group and in 62.0% of the non-CP-A/PS-0/1 group.

At radiological PD during Atz+Bev therapy, Child–Pugh scores were 5 (*n* = 46), 6 (*n* = 31), 7 (*n* = 10), 8 (*n* = 4), 9 (*n* = 4), and 10 (*n* = 2); overall, 77 patients (79.4%) remained in Child–Pugh class A. ECOG PS was 0 in 53 patients, 1 in 34, 2 in 9, and 3 in 1, with 87 patients (89.7%) maintaining PS 0/1. A total of 76 patients (78.4%) met both criteria (CP-A/PS-0/1 group), while 21 (21.6%) did not (non-CP-A/PS-0/1 group) (Figure 3).

### 3.5. Prognostic Factors Associated with PPS at PD

Prognostic factors associated with PPS at PD are shown in Table 2. On univariate analysis, ECOG PS 0, Child–Pugh A, modified albumin–bilirubin (mALBI) grade of 1 or 2a, BCLC stage B, absence of portal vein invasion, AFP < 400 ng/mL, and DC at 6 weeks by Atz+Bev therapy were significantly associated with longer PPS. On multivariate analysis, ECOG PS 0 (hazard ratio [HR] 0.438, 95% CI 0.246–0.782; *p* = 0.0052), Child–Pugh A (HR 0.307, 95% CI 0.145–0.650; *p* = 0.0021), absence of portal vein invasion (HR 0.460, 95% CI 0.222–0.951; *p* = 0.0363), and AFP < 400 ng/mL (HR 0.557, 95% CI 0.316–0.980; *p* = 0.0426) remained independent predictors of longer PPS.

### 3.6. Baseline Factors Associated with Preserving CP-A/PS-0/1 Status at PD

Baseline factors associated with maintaining CP-A/PS-0/1 status at PD are summarized in Table 3. On univariate analysis, a baseline Child–Pugh score of 5, mALBI grade of 1, and BCLC stage A or B were significantly associated with preservation of CP-A/PS-0/1. On multivariate analysis, only a baseline Child–Pugh score of 5 independently predicted maintaining CP-A/PS-0/1 status (odds ratio [OR] 5.882, 95% CI 1.733–20.00; *p* = 0.0045).

### 3.7. Transition to and Type of Subsequent Treatment After PD

Among the 76 patients classified as CP-A/PS-0/1 at PD, 71 (93.4%) transitioned to subsequent therapy, whereas only 8 of the 21 patients (38.0%) in the non-CP-A/PS-0/1 group received further anticancer treatment. The remaining 5 patients (6.6%) and 13 patients (62.0%), respectively, received BSC alone (Figure 3). In the CP-A/PS-0/1 group, the most frequently used regimens were lenvatinib (*n* = 25), ramucirumab (*n* = 12), continuation or resumption of Atz+Bev (*n* = 12), durvalumab plus tremelimumab (*n* = 6), and cabozantinib (*n* = 5). In the non-CP-A/PS-0/1 group, treatment options were limited: four patients received ramucirumab, two received lenvatinib, one received cabozantinib, and one underwent TACE.

### 3.8. Antitumor Response to Subsequent Treatment

Best overall antitumor responses after radiological progression on Atz+Bev in the entire cohort and stratified by clinical condition at progression are summarized in Table 4. In the CP-A/PS-0/1 group, the ORR and DCR were 15.8% and 72.4%, respectively. In contrast, in the non-CP-A/PS-0/1 group, the ORR was 4.8%, and the DCR was markedly lower at 14.3%. While the difference in ORR was not statistically significant (*p* = 0.2867), the difference in DCR was highly significant (*p* < 0.0001). In a sensitivity analysis, among patients who actually received subsequent anticancer therapy after progression (*n* = 79), the best overall responses were CR/PR/SD/PD/NE = 0/13/45/14/7. DC (PR/SD) was achieved in 58 patients, whereas 21 patients were classified as non-DC (PD/NE).

### 3.9. PPS After PD on Atz+Bev

**Figure 4:**
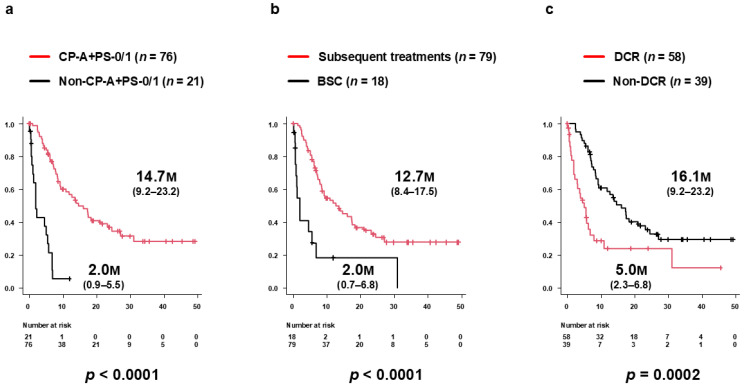
Subgroup analyses of post-progression survival (PPS) after PD during Atz+Bev therapy. (**a**) PPS stratified by clinical status at PD. Patients in the CP-A/PS-0/1 group (*n* = 76) had significantly longer PPS (median 14.7 months; 95% CI, 9.2–23.2) than those in the non-CP-A/PS-0/1 group (*n* = 21; 2.0 months; 95% CI, 0.9–5.5; *p* < 0.0001). (**b**) PPS according to receipt of subsequent therapy. Patients who received further therapy had significantly longer PPS (median 12.7 months; 95% CI, 8.4–17.5) than those managed with BSC alone (2.0 months; 95% CI, 0.7–6.8; *p* < 0.0001). (**c**) PPS according to best overall response during subsequent therapy. Patients who achieved disease control (DC) experienced significantly longer PPS (16.1 months; 95% CI, 9.2–23.2) than those without DC (5.0 months; 95% CI, 2.3–6.8; *p* = 0.0002).

Median PPS among patients who developed PD during Atz+Bev therapy was 9.2 months (95% CI 7.0–16.1 months) (Figure 2). Median PPS was significantly longer in the CP-A/PS-0/1 group (14.7 months, 95% CI 9.2–23.2 months) than in the non-CP-A/PS-0/1 group (2.0 months, 95% CI 0.9–5.5 months; *p* < 0.0001) (Figure 4a). Similarly, PPS was longer in patients who received subsequent therapy (12.7 months, 95% CI 8.4–17.5 months) compared with those receiving BSC alone (2.0 months, 95% CI 0.7–6.8 months; *p* < 0.0001) (Figure 4b). Further, patients who achieved DC as the best overall response during subsequent therapy had significantly longer PPS (16.1 months, 95% CI 9.2–23.2 months) than those without DC (5.0 months, 95% CI 2.3–6.8 months; *p* = 0.0002) (Figure 4c). In a sensitivity analysis restricted to patients who received subsequent anticancer therapy, PPS remained significantly longer in the DC group than in the non-DC group (median, 16.1 months [95% CI, 9.2–23.2] vs. 5.7 months [95% CI, 3.2–NR]; log-rank *p* = 0.0306).

## 4. Discussion

This study evaluated PPS and its clinical determinants in patients with advanced HCC treated with Atz+Bev, focusing on liver function and PS at treatment initiation and at radiological progression. Maintaining Child–Pugh class A and ECOG PS 0/1 at radiological progression was associated with longer PPS. A baseline Child–Pugh score of 5 independently predicted preservation of this favorable clinical status at progression. These findings support a “two-timepoint” strategy: initiate Atz+Bev under optimal hepatic reserve (Child–Pugh 5) and prioritize its preservation through progression to maintain eligibility for subsequent therapy and thereby prolong PPS.

Our findings are consistent with observations from the tyrosine kinase inhibitor era, in which PPS strongly depended on liver function and PS at progression [11,12]. Moreover, our prior sorafenib-era data similarly suggested that a baseline Child–Pugh score 5 facilitates the preservation of hepatic reserve at progression and subsequent treatment transition [13]. A post hoc analysis of IMbrave150 also indicated that the OS advantage of Atz+Bev over sorafenib was more apparent in patients with better liver function [14]. Collectively, these data emphasize that maintaining hepatic reserve remains a central prerequisite for sustained systemic therapy even in the immunotherapy era.

In the present analysis, we evaluated liver function at radiological progression using the ALBI grade. While a favorable mALBI grade (1/2a) was associated with a longer PPS in univariate analysis, it did not retain independent prognostic significance after adjustment for Child–Pugh class and PS. As expected, portal vein invasion—an established adverse prognostic factor in HCC [1]—remained independently associated with PPS, underscoring that post-progression outcomes reflect not only functional status but also tumor aggressiveness.

Clinically, these findings underscore the importance of proactive management to preserve hepatic reserve and PS throughout Atz+Bev therapy, including early identification and prompt management of treatment-related adverse events and decompensation risks. Conversely, patients who become ineligible for further therapy due to functional decline at progression remain a major clinical challenge. Future studies should develop optimized strategies and predictive tools for this population, including models integrating hepatic function dynamics and tumor factors [15].

Several limitations warrant consideration. This retrospective, single-center design is subject to residual confounding and selection bias, and post-progression treatment was not standardized. In addition, given the limited sample size within each subsequent treatment category, we did not perform formal regimen-specific comparative analyses, which could be underpowered and potentially misleading; instead, our results emphasize liver function and PS as upstream determinants enabling treatment sequencing across heterogeneous real-world strategies. Moreover, because we restricted the cohort to patients with Child–Pugh class A and ECOG PS 0/1 at baseline, the applicability of our findings to patients with poorer liver function or performance status remains uncertain. Due to the retrospective clinical nature of this study and limited availability of biospecimens, we could not explore the molecular or immunological mechanisms underlying preservation of treatment eligibility. Finally, because the cohort consisted exclusively of Japanese patients, external validation in more diverse populations is required.

## 5. Conclusions

In patients with advanced HCC treated with Atz+Bev, longer PPS after radiological progression was primarily associated with preservation of Child–Pugh A and ECOG PS 0/1 at progression. Initiating Atz+Bev under optimal baseline liver function, particularly at a Child–Pugh score of 5, increased the likelihood of maintaining these favorable conditions, thereby enabling subsequent therapy and improving post-progression outcomes.

## Figures and Tables

**Table 1:** Baseline characteristics at initiation of Atz+Bev in all patients and those with radiologically confirmed progressive disease (PD).

Characteristics at Initiation of Atz+Bev	All Patients (*n* = 132)	Radiologically Confirmed PD Patients (*n* = 97)
Age, median (range), years	75 (38–90)	74 (38–90)
Sex (men/women), *n*	109/23	80/17
Etiology (HBV/HCV/non-viral), *n*	17/26/89	15/19/63
ECOG PS (0/1), *n*	105/27	75/22
Child-Pugh score (5/6), *n*	96/36	69/28
mALBI grade (1/2a/2b), *n*	52/42/38	38/33/26
BCLC stage (A/B/C), *n*	3/61/68	3/45/49
Number of tumors (<4/≥4), *n*	39/93	29/68
Tumor size (<50/≥50 mm), *n*	83/49	66/31
Portal vein invasion (−/+), *n*	107/25	79/18
Extrahepatic metastasis (−/+), *n*	81/51	61/36
AFP, median (range), ng/mL	39.7 (1.8–2,037,310)	46.3 (1.9–233,543)
AFP level (<400/≥400 ng/mL), *n*	97/35	72/25
DCP, median (range), mAU/mL	595.5 (10–403,328)	613 (10–115,481)
DCP level (<400/≥400 mAU/mL), *n*	60/72	42/55
NLR, median (range)	2.47 (0.59–14.19)	2.56 (0.59–14.19)
Treatment line (1st/2nd/3rd/4th), *n*	94/36/1/1	63/32/1/1
Observation period, median (range), months	16.1 (0.8–53.6)	18.6 (1.4–53.6)

Abbreviations: Atz+Bev, atezolizumab plus bevacizumab; PD, progressive disease; HBV, hepatitis B virus; HCV, hepatitis C virus; ECOG PS, Eastern Cooperative Oncology Group performance status; mALBI, modified albumin–bilirubin; BCLC, Barcelona Clinic Liver Cancer; AFP, alpha-fetoprotein; DCP, des-γ-carboxy prothrombin; NLR, neutrophil-to-lymphocyte ratio.

**Table 2:** Univariate and multivariate analyses of prognostic factors for post-progression survival (PPS) in patients who experienced radiologically confirmed progression during Atz+Bev therapy (*n* = 97).

	Univariate Analysis		Multivariate Analysis	
Factors	HR (95% CI)	*p* Value	HR (95% CI)	*p* Value
Age (≥75 years)	0.797 (0.483–1.314)	0.3736	−	−
Sex (female)	0.610 (0.290–1.284)	0.1930	−	−
ECOG PS (0)	0.413 (0.249–0.684)	0.0006	0.438 (0.246–0.782)	0.0052
Child–Pugh (A)	0.152 (0.081–0.285)	<0.0001	0.307 (0.145–0.650)	0.0021
mALBI grade (1/2a)	0.435 (0.257–0.735)	0.0019	0.544 (0.291–1.020)	0.0575
BCLC stage (B)	0.531 (0.319–0.883)	0.0147	0.901 (0.479–1.698)	0.7479
Portal vein invasion (−)	0.527 (0.295–0.940)	0.0299	0.460 (0.222–0.951)	0.0363
Extrahepatic metastasis (−)	0.603 (0.362–1.002)	0.0510	−	−
AFP (<400 ng/mL)	0.521 (0.313–0.867	0.0120	0.557 (0.316–0.980)	0.0426
DCP (≥400 mAU/mL)	1.309 (0.765–2.241)	0.3257	−	−
DC at 6 weeks by Atz+Bev (RECIST v1.1) (+)	0.506 (0.295–0.868)	0.0132	0.628 (0.337–1.171)	0.1435

Abbreviations: PPS, post-progression survival; Atz+Bev, atezolizumab plus bevacizumab; HR, hazard ratio; CI, confidence interval; ECOG PS, Eastern Cooperative Oncology Group performance status; mALBI, modified albumin–bilirubin; BCLC, Barcelona Clinic Liver Cancer; AFP, alpha-fetoprotein; DCP, des-γ-carboxy prothrombin; DC, disease control; RECIST v1.1, Response Evaluation Criteria in Solid Tumors version 1.1.

**Table 3:** Baseline factors at initiation of Atz+Bev associated with maintaining Child–Pugh A and ECOG PS 0/1 status at the time of radiologically confirmed progressive disease (PD).

	Univariate Analysis		Multivariate Analysis	
Factors	OR (95% CI)	*p* Value	OR (95% CI)	*p* Value
Age (<75 years)	0.384 (0.139–1.058)	0.0642	–	–
Sex (male)	1.140 (0.329–3.949)	0.8359	–	–
ECOG PS (1)	0.492 (0.169–1.432)	0.1931	–	–
Etiology (non-viral)	0.686 (0.239–1.970)	0.4835	–	–
Child–Pugh score (5)	8.850 (3.021–26.32)	<0.0001	5.882 (1.733–20.00)	0.0045
mALBI grade (1)	5.128 (1.391–18.87)	0.0140	2.079 (0.458–9.434)	0.3431
BCLC stage (A/B)	3.086 (1.081–8.850)	0.0352	2.538 (0.806–8.000)	0.1114
Tumor size (<50 mm)	0.601 (0.198–1.824)	0.3687	–	–
Number of tumors (<4)	2.083 (0.634–6.847)	0.2266	–	–
Portal vein invasion (−)	2.133 (0.689–6.604)	0.1887	–	–
Extrahepatic metastasis (+)	0.735 (0.275–1.965)	0.5390	–	–
AFP (≥400 ng/mL)	1.611 (0.564–4.605)	0.3734	–	–
DCP (≥400 mAU/mL)	0.626 (0.237–1.654)	0.3448	–	–
NLR (<2.36)	2.024 (0.709–5.779)	0.1879	–	–
Treatment line (2nd or later)	0.654 (0.243–1.755)	0.3988	–	–

Abbreviations: Atz+Bev, atezolizumab plus bevacizumab; OR, odds ratio; CI, confidence interval; ECOG PS, Eastern Cooperative Oncology Group performance status; mALBI, modified albumin–bilirubin; BCLC, Barcelona Clinic Liver Cancer; AFP, alpha-fetoprotein; DCP, des-γ-carboxy prothrombin; NLR, neutrophil-to-lymphocyte ratio.

**Table 4:** Best overall antitumor response after progression on Atz+Bev according to RECIST v1.1 in patients with radiologically confirmed progressive disease (PD) (*n* = 97).

RECIST v1.1	All Patients (*n* = 97)	CP-A+PS-0/1 (*n* = 76)	Non-CP-A+PS-0/1 (*n* = 21)	*p* Value
CR/PR/SD/PD/NE, n	0/13/45/14/25	0/12/43/12/9	0/1/2/2/16	–
ORR	13.4%	15.8%	4.8%	0.2867
DCR	59.8%	72.4%	14.3%	<0.0001

Abbreviations: Atz+Bev, atezolizumab plus bevacizumab; RECIST, Response Evaluation Criteria in Solid Tumors; CR, complete response; PR, partial response; SD, stable disease; PD, progressive disease; NE, not evaluable; ORR, objective response rate; DCR, disease control rate; CP-A, Child–Pugh A; PS, performance status. Note: response assessments included patients who received subsequent anticancer therapy as well as those managed with best supportive care.

## Data Availability

The data presented in this study are available on request from the corresponding author. The data are not publicly available due to privacy and institutional restrictions.

## Abbreviations

The following abbreviations are used in this manuscript:

AE	adverse event
AFP	alpha-fetoprotein
ALBI	albumin–bilirubin
Atz+Bev	atezolizumab plus bevacizumab
BCLC	Barcelona Clinic Liver Cancer
BSC	best supporting care
CI	confidence interval
CP-A	Child–Pugh A
CR	complete response
DC	disease control
DCR	disease control rate
DCP	des-gamma-carboxy prothrombin
ECOG	Eastern Cooperative Oncology Group
HBV	hepatitis B virus
HCC	hepatocellular carcinoma
HCV	hepatitis C virus
HR	hazard ratio
ICI	immune checkpoint inhibitor
mALBI	modified albumin–bilirubin
NE	not evaluable
NLR	neutrophil-to-lymphocyte ratio
OR	objective response
ORR	objective response rate
OS	overall survival
PD	progressive disease
PFS	progression-free survival
PPS	post-progression survival
PR	partial response
PS	performance status
RECIST	Response Evaluation Criteria in Solid Tumors
SD	stable disease
TACE	trans-arterial chemoembolization
